# The complete chloroplast genome of *Aconitum scaposum*

**DOI:** 10.1080/23802359.2021.1944380

**Published:** 2021-06-28

**Authors:** Min Zhang, Jiawei Luo, Lijun Su, Qiaojiao Ding, Xianmei Yin, Feixia Hou, Jihai Gao, Cheng Peng

**Affiliations:** Key Laboratory of Distinctive Chinese Medicine Resources in Southwest China, Pharmacy College, Chengdu University of Traditional Chinese Medicine, Chengdu, China

**Keywords:** *Aconitum scaposum*, chloroplast genome, phylogenetic tree

## Abstract

*Aconitum scaposum* Franch 1894 belongs to the Genus *Aconitum* and Subgenus *Lycoctonum* (Ranunculaceae). It is widely distributed in China and adjacent areas, used as herbal medicine and had highy toxic components. This species has little reasearch information, especially its chloroplast (cp) genome information being unclear. Therefore, with the method of high salt and low pH to extract the cp of *A. scaposum*, we sequenced and assembled the complete cp genome of *A. scaposum* using Illumina high-throughput sequencing platform. The results showed the cp genome of *A. scaposum* was 157 688 bp in length, including a pair of inverted repeated regions (IRa 26 156 bp and IRb 26 232 bp, respectively), large single copy region (LSC 69 309 bp) and small single copy region (SSC 16 917 bp). And cp genome of *A. scaposum* consisted of 145 unique genes, 8 ribosomal RNA (rRNA) genes and 38 transfer RNA (tRNA) genes, with GC content was 38%. Meanwhile, based on the cp complete genome, we performed the phylogenetic tree of **66** species with maximum likelihood (ML) method, respectively. Among them, we selected one *Delphinium* species as the outgroup and the bootstrap of each braches were greater than 90%. The results indicated that the phylogenetic relationship of *A. scaposum* was **relatively closely related to *A. scaposum* var. *vaginatum* compared to other *Aconitum* species**.

*Aconitum* is a kind of important medicinal plant that are well-known in many Aisan countries, notably China, India and Japan (Liu et al. 2017; Kong et al. 2017). *Aconitum* is divided into three subgenera: Ranunculaceae, Aconitum and Gymnaconitum (Inkyu 2017). The lateral root of *Aconitum* has the effects on cardiovascular system, anti-inflammation, dispelling wind damp and et al (Zhou et al. 2015). And *Aconitum* is mainly used for analgesia and anti-inflammatory purposes (Meng et al. 2018). At present, There are about over 200 species of *Aconitum* in China (Liu et al. 2020), especially the species of *Aconitum scaposum* Franch 1894. Here, we assembled and characterized the cp genome of *A. scaposum* using Illumina sequencing data, providing a valuable genomic resource for future studies.

Fresh leaves material of *A. scaposum* were selected from Sichuan Province, China (103.83°E, 30.70°N), and a specimen and DNA sample were deposited in the Herbarium of Chengdu University of Traditional Chinese Medicine (http://grb.cdutcm.edu.cn/, Gao Jihai, gaojihai@cdutcm.edu.cn) under the voucher number 2151C0001100003858 and 51011520191010GJH0031, respectively. CpDNA was extraced by a combination of high salt and low pH method (Li et al. 2017) and modified CTAB method (Attitalla 2011). Then, the library was constructed and the cpDNA was sequenced by Illumina HiSeq PE14. The clean reads were obtained by de novo assembly from whole-genome sequence data by abyss 2.0 software (Jackman et al. 2017). Among them, we selected *A. sinomontanum* as the reference sequence. The gene annotation was perforemed using the online program CpGAVAS2 (Conant and Wolfe 2008). The cp genome together with gene annotations was submitted to GenBank under the accession number of MW817090.

In order to explore the phylogenic relationships of the partial species among *Aconitum*, **66 **cp complete genomes were selected to construct phylogenetic tree by ML method, respectively. Moreover, **66** cpDNA sequences were aligned using mafft align (Katoh and Standley 2013) and the phylogenic tree was performed in megaX (Lu et al. 2020), with a bootstrap of 1000 replicates.

The cp complete genome of *A. scaposum* is 157 688 bp, including LSC region of 69 309 bp, SSC region of 16 917 bp. The GC content of *A. scaposum* was 38%. In addition, we annotated 145 genes, including 38 tRNA genes and 8 rRNA genes. In order to further study the phylogeny of *Aconitum*, the phylogenetic tree of **66** species with ML method (Figure 1) indicated ***A. scaposum* was relatively closely related to *A. scaposum* var. *vaginatum* compared to other *Aconitum* species**, with over 90% bootstap support. **This result will provide valuable insight into conservation and evolutionary histories for *A. scaposum*.**

**Figure 1.**
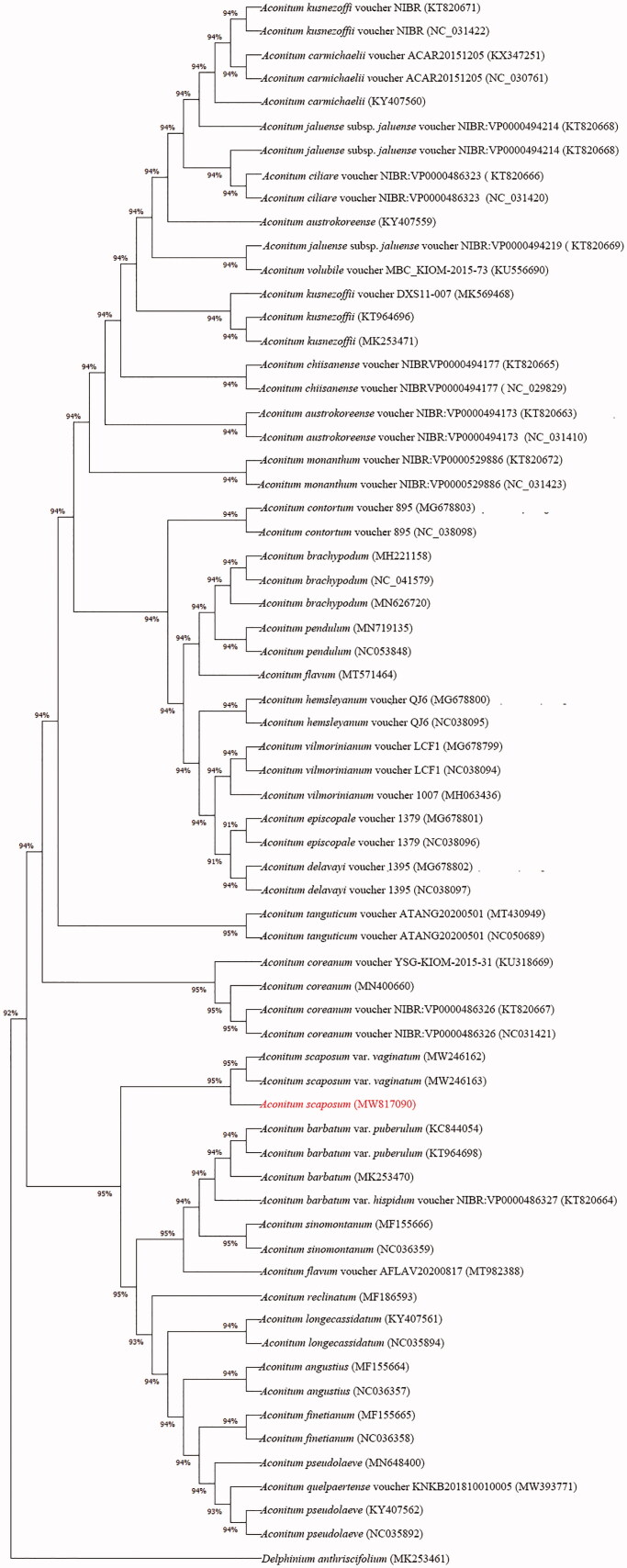
Phylogenetic relationships of *Aconitum* species using whole chloroplast genome.

## Data Availability

The genome sequence data that support the findings of this study are openly available in NCBI at http://www.ncbi.nlm.nih.gov/ under the accession number [MW817090], or available from the corresponding author. The associated BioProject, SRA, and Bio-Sample numbers are PRJNA717893, SRR14085846, and SAMN18515267 respectively.”
